# Localization of putative binding sites for cyclic guanosine monophosphate and the anti-cancer drug 5-fluoro-2′-deoxyuridine-5′-monophosphate on ABCC11 *in silico* models

**DOI:** 10.1186/1472-6807-13-7

**Published:** 2013-05-06

**Authors:** Mylène Honorat, Raphaël Terreux, Pierre Falson, Attilio Di Pietro, Charles Dumontet, Lea Payen

**Affiliations:** 1 INSERM, UMR-S1052, Centre de Recherche en Cancérologie de Lyon, Lyon F-69008, France; 2 CNRS, UMR 5286, Centre de Recherche en Cancérologie de Lyon, Lyon F-69008, France; 3 Université Lyon 1, UMR 1052, Centre de Recherche en Cancérologie de Lyon, Lyon F-69008, France; 4 Centre Léon Bérard, Lyon F-69008, France; 5 LabEx DEVweCAN, Lyon F-69008, France; 6 Institut des Sciences Pharmaceutiques et Biologiques, Université Lyon 1, Lyon F-69008, France; 7 CNRS, UMR 5086, Institut de Biochimie et Chimie des Protéines, BMSSI, Lyon F-69007, France; 8 Université Lyon 1, UMR 5086, Lyon F-69007, France; 9 Hospices Civils de Lyon, Centre hospitalier de biologie sud, Laboratoire de biochimie et biologie moléculaire, Pierre-Bénite F-69310, France; 10 Hospices Civils de Lyon, Centre hospitalier de biologie sud, Laboratoire de biochimie, Pierre-Bénite F-69310, France

**Keywords:** ABC transporter, ABCC11/MRP8, cGMP, 5FdUMP, Homology modelling, Docking, Dynamics simulation

## Abstract

**Background:**

The Multidrug Resistance Protein ABCC11/MRP8 is expressed in physiological barriers and tumor breast tissues in which it secretes various substrates including cGMP (cyclic guanosine monophosphate) and 5FdUMP (5-fluoro-2′-deoxyuridine-5′-monophosphate), the active metabolite of the anticancer drug 5-FluoroUracil (frequently included to anticancer therapy).

Previously, we described that ABCC11 high levels are associated to the estrogen receptor (ER) expression level in breast tumors and in cell lines resistant to tamoxifen. Consequently, by lowering the intracellular concentration of anticancer drugs, ABCC11 likely promotes a multidrug resistance (MDR) phenotype and decreases efficiency of anticancer therapy of 5FdUMP. Since no experimental data about binding sites of ABCC11 substrate are available, we decided to *in silico* localize putative substrate interaction sites of the nucleotide derivatives. Taking advantage of molecular dynamics simulation, we also analysed their evolution under computational physiological conditions and during the time.

**Results:**

Since ABCC11 crystal structure is not resolved yet, we used the X-ray structures of the mouse mdr3 (homologous to human ABCB1) and of the bacterial homolog Sav1866 to generate two independent ABCC11 homology models in inward- and outward-facing conformations. Based on docking analyses, two putative binding pockets, for cGMP and 5FdUMP, were localized in both inward- and outward-facing conformations. Furthermore, based on our 3D models, and available biochemical data from homologous transporters, we identified several residues, potentially critical in ABCC11 transport function. Additionally, molecular dynamics simulation on our inward-facing model revealed for the first time conformation changes assumed to occur during transport process.

**Conclusions:**

ABCC11 would present two binding sites for cGMP and for 5FdUMP. Substrates likely first bind at the intracellular side of the transmembrane segment while ABCC11 is open forward the cytoplasm (inward-facing conformation). Then, along with conformational changes, it would pass through ABCC11 and fix the second site (close to the extracellular side), until the protein open itself to the extracellular space and allow substrate release.

## Background

ABCC11/MRP8 (Multidrug Resistance protein 8) is a human ABC (ATP-Binding Cassette) transporter that secretes several endogenous substrates [1-3] including cAMP, cGMP, as well as exogenous-derived molecules [1-7] such as 5FdUMP (5′-fluoro-2′-deoxyuridine monophosphate), the active metabolite of 5-FluoroUracil, methotrexate and aracytine. ABCC11 is expressed in various tissues such as physiological barriers, suggesting a role in body detoxification [3,8,9]. Its expression was associated with a decrease of the clinical response to breast tumor chemotherapy [10] and with a low probability of survival in acute myeloid leukemia [7]. Furthermore, ABCC11 is overexpressed in estrogen receptor-positive cell lines resistant to tamoxifen [5] and in ERBB2-overexpressing breast tumors [11]. By decreasing retention of anticancer agent in cells, ABCC11 contributes to a multidrug resistance (MDR) phenotype leading to cell resistance to a broad range of anticancer agents.

Transport of cGMP by ABCC11 was largely described by Guo et al. in 2003. They compared the cGMP intracellular contents of ABCC11 overexpressing cells to their counterpart (cells transfected with empty vector). Only, the ABCC11 positive cells could extrude cGMP under stimulation by SIN-1A. Additionally, they described 5FdUMP transport by ABCC11 in inside-out membrane vesicles. Vesicles bearing high levels of ABCC11 showed an increased accumulation of 5FdUMP and allow to conclude that 5FdUMP is an ABCC11 substrate. Nevertheless, no pharmacokinetic study was undertaken to determine kinetic parameters (Km and Vm values) neither for cGMP, nor for 5FdUMP. For the ABCC4 homolog the Km of cGMP was 180 ± 20 μM [12].

ABCC11 is predicted to contain two Membrane-Spanning Domains (MSDs) and two Nucleotide-Binding Domains (NBDs) in a common MSD1-NBD1-MSD2-NBD2 topology with intracellular N- and C-termini. Each MSD is constituted by six transmembrane segments (TMs). Since this protein has not yet been crystallized, and confident algorithm tools have been developed to generate homology models based on reference templates [13], we generated two *in silico* models. Docking analyses led us to identify several amino acid residues potentially implicated in ABCC11 transport activity. Additionally, molecular dynamics simulation revealed for the first time conformational changes that would occur during the transport process.

## Results and discussion

### ABCC11 inward- and outward-facing models

**Figure 1:**
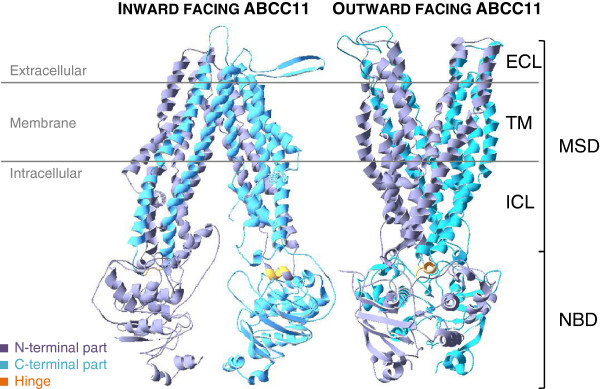
**Mouse ABCB1- and Sav1866-based models of ABCC11 respectively adopt inward- and outward-facing conformations.** ECL: Extracellular Loop; TM: Transmembrane segment; ICL: Intracellular Loop; MSD: Membrane spanning domain; NBD: Nucleotide binding domain.

The first model was based on the mouse mdr3 X-ray structure [14] (Figure 1). Mouse mdr3 which shares 87% homology with the human protein (ABCB1) was crystallized in a nucleotide-free inward-facing conformation. Our ABCC11 model adopts, as expected, an inward-facing conformation where the assumed drug-binding pocket is exposed to the intracellular space, allowing drug binding before NBDs dimerization in the presence of ATP (Figure 1). This conformation showed distant NBDs and transmembrane helices bundled next to the extracellular side of the membrane.

**Figure 2:**
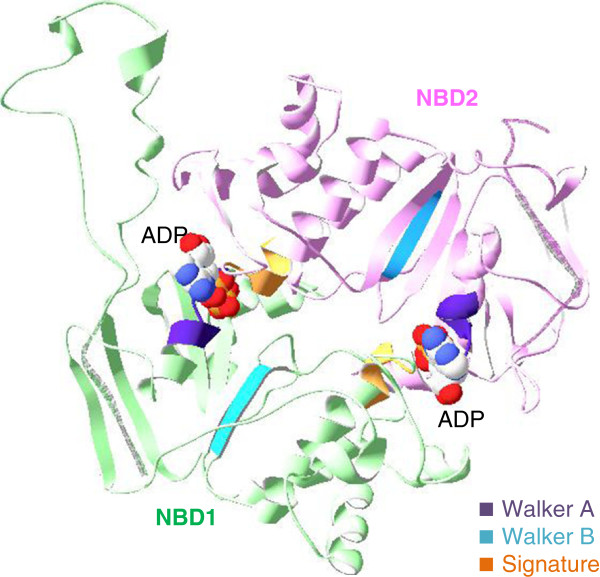
**The ADP molecules are sandwiched between the two NBDs of ABCC11.** ADP molecules (ball-shape) are inserted between the Walker A sequence of one NBD (purple) and the signature sequence of the other NBD (orange).

The second 3D model was based on the Sav1866 crystal structure from *Staphylococcus aureus* obtained in the presence of ADP, and adopted an outward-facing conformation [15,16]. Compared to the first model, the conformation is logically reversed (Figure 1); the substrate cavity is exposed to the extracellular space allowing release of bound substrates. In this conformation, ATP hydrolysis and ADP release are expected to bring the transporter back to an inward-facing conformation. This outward-facing conformation presents the TM helices bundled next to the NBDs, closing the access from the cytoplasmic side of transmembrane domains. The NBDs are associated inside a dimer in which nucleotides are sandwiched (Figure 2).

In both ABCC11 models, MSD helices extend from membrane to cytoplasm, forming two intracellular loops (ICLs) in each MSD. The first one is in contact with the *cis*-NBD while the second one makes contacts with the *trans*-NBD through its “hinge” sequence. By contrast to mouse ABCB1 that showed similar hinge sequences (RTVI in MSD1 and RTVV in MSD2), ABCC11 does not display any conserved hinge sequences with KLIK in MSD1 and SSIH in MSD2. Such a contact is supposed to transduce conformational changes occurring from NBD to MSD.

**Figure 3:**
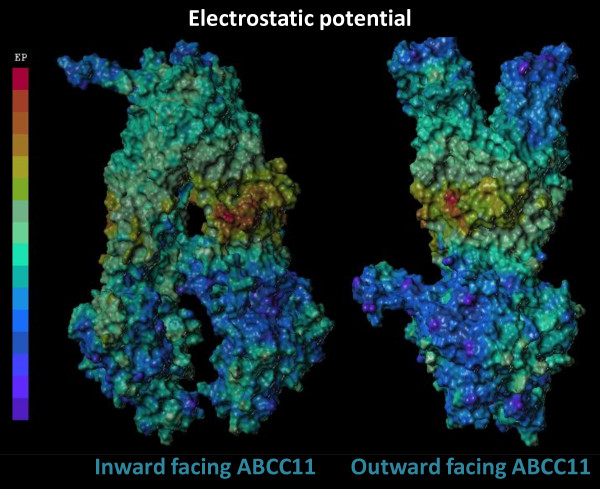
**Electrostatic potential of ABCC11 models.** Electrostatic potentials are represented with a scale from warm (positive charges) to cold (negative charges) colors.

The ICLs show an interesting positively-charged region at the membrane leaflet (Figure 3) containing several lysine and arginine residues. These positive charges, just under the intracellular membrane side, were likely involved in the drug transport process. This may also influence the accurate protein inclusion into the membrane and enhance its stability as described for ABCC1 [17]. Indeed, Arg235, Lys988, Arg989 and Arg1044 of ABCC11 respectively corresponded to Lys396, Lys1141, Arg1142 and Arg1197 of ABCC1 [18-20] that were described to be functionally important in organic anion transport. These residues might be involved in ABCC11-mediated substrate transport. The ABCC11 substrate-translocation site was essentially positively charged, consistent with its ability to transport anions. A similar observation was made for its ABCC5 homolog [21].

### cGMP and 5FdUMP binding sites in ABCC11 models

**Figure 4:**
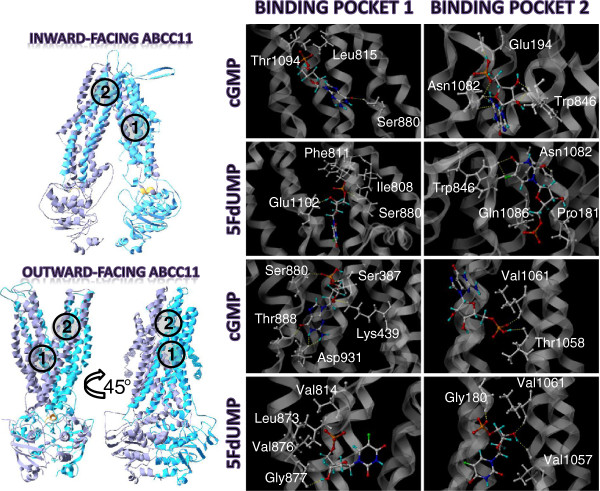
**cGMP and 5FdUMP binding to the two putative binding pockets.** The best positions revealed vicinal residues (grey) and putative hydrogen bonds (yellow) between substrates and residues.

**Figure 5:**
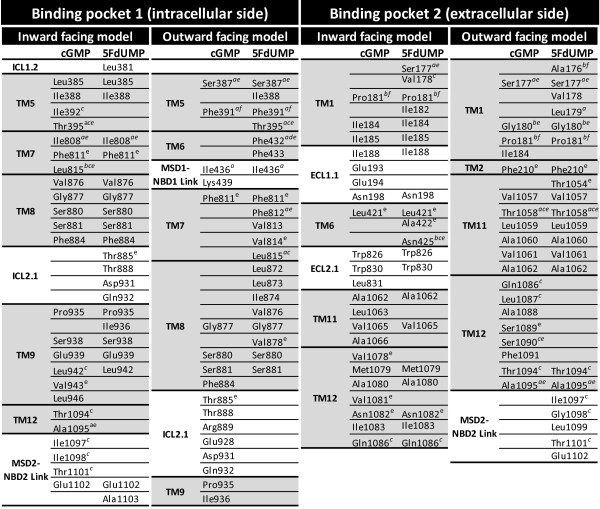
**ABCC11 residues in a 5 Å diameter around bound ligand**. **a**) Residues described in the putative binding pocket of ABCC5 (Ravna et al.). **b**) Residues described in the putative binding pocket of ABCC5 and conserved in ABCC11 (Ravna et al.). **c**) Residues described in the binding pocket of ABCB1 (Aller et al.). **d**) Residues described in the binding pocket of ABCB1 and conserved in ABCC11 (Aller et al.). **e**) Residues described in the putative binding pocket of ABCC4 (Ravna et al.). **f**) Residues described in the putative binding pocket of ABCC4 and conserved in ABCC11 (Ravna et al.).

Docking analysis on our ABCC11 models revealed two putative binding pockets for cGMP and 5FdUMP (Figure 4). An internal binding site (named pocket 1) in ABCC11 was located next to the intracellular side of the transmembrane region. It involved TM5, 7, 8, 9 and 12 (Figure 5). In this pocket, cGMP interacted with Ser880 in the inward-facing conformation of ABCC11, and with Ser387, Lys439, Ser880, Thr888 and Asp931 in the outward-facing model. In the inward-facing ABCC11 model, 5FdUMP was in interaction with Ser880 and Glu1102, while in the outward-facing model, it established only one hydrogen bond with Gly877.

An external ABCC11 binding pocket (named pocket 2) was located close to the extracellular space, and formed by TM1, 6, 11 and 12 (Figure 5). In the inward-facing ABCC11 model, cGMP could interact with Glu194, Asn1082 and Trp826 while, in the outward-facing conformation, only two hydrogen bonds were observed with Thr1058 and Val1061, suggesting less interactions. 5FdUMP could interact with Trp826, and Asn1082, in the inward-facing model while Gly180, Val1057 and Val1061 were involved in interactions within the outward-facing model.

The local similarity does not exceed 24.3% between binding sites of ABCC11 and the correspondent residues in mABCB1. Between binding sites of ABCC11 and the correspondent residues in SAV1866, similarity does not go over 29.7%. The similarity figures are sufficient but lower than those between ABCC11 and its ABCC4 homolog for example (until 43.2% of similarity). This suggests that homology modeling permits the establishment of models respecting the binding pocket structure and thus the specificity of each protein for their substrate spectrum. This point supports the fact that docking experiments are reliable on these models of ABCC11.

The identification of two potential binding pockets strongly suggests that ligands can bind to two different sites. Indeed, the QZ59-SSS cyclic peptide inhibitor showed two different binding sites in mouse mdr3 X-ray structure [14]. Based on docking analyses, MK571 (a powerful inhibitor of many ABCCs including ABCC11) also bound efficiently to both sites *in silico* (data not shown). This observation was strongly supported by the fact that MK571 is a competitive inhibitor of ABCC1-mediated LTC4 transport [22]. MK571 binding to one site would then not permit substrate transport. Currently, no experimental data allows us to know if ABCC11 substrates bind either simultaneously to both sites, randomly to each site, or preferentially to the internal or external site.

### ABCC11 residues potentially involved in substrate transport

Because of their spatial position and putative interaction with ligands, many of the residues reported in Figure 5 might be important for ligand binding. Some residues might directly interact with substrate while others might be involved in allosteric modifications of ABCC11 conformation and affect the substrate binding affinity. Some of the residues constituting ABCC11 pockets corresponded to residues described for ABCC5 (Figure 5), the closest ABCC member of ABCC11 [23]. Indeed, Ravna et al. also described two putative binding pockets for cGMP. The first binding pocket of ABCC11, involving TM5, 7, 8, 9 and 12, would correspond to one of the two binding pockets identified in ABCC5 (also involving TM5, 7 and 8). The second ABCC11 binding pocket was formed by TM1, 6, 11 and 12, similarly to the second binding pocket described in ABCC5 [23]. ABCC11 Thr1058 (TM11) was located inside a binding site as well as its equivalent residues in ABCC5 (Ile1107 [23]) and mouse ABCB1 (Tyr949 [14,24]). Some residues (Ala176, Pro181, Leu815, Asn425) found in the binding pockets are conserved between the two cGMP transporters, suggesting their putative function in drug binding and/or transport.

In the same manner, several residues identified in ABCC11 substrate pockets also correspond to residues found in the substrate translocation chamber of ABCC4 outward facing model of Ravna et al. (Figure 5) [25]. Three of them are conserved between ABCC4 and ABCC11 (ABCC11 residues Ala176, Pro181, Phe391). The majority of those residues were located in binding pockets of the outward facing model of ABCC11. As listed in Figure 5, several ABCC11 residues identified near the bound ligand also have equivalent residues in the QZ59-binding pocket of mouse ABCB1. In addition, Ser1090 corresponds to both ABCC1 Tyr1243 [26] and ABCB1 Val982 [27] described to be important for the transport function.

Among those residues, it is important to note that Thr395, Phe432, Asn425, Leu815 and Thr1058 have been described in ABCB1, ABCC4 and ABCC5 putative substrate binding pockets. And finally, some residues found in the drug-binding pockets have equivalents in other ABC transporter that have been described to be essential for transport function: Ile198 (ABCC1 Lys347 [18]), Phe433 (ABCC4 Phe 368 [28]), Lys439 (ABCC4 Glu374 [28]), Pro935 (ABCB1 Gly830 [29]), Ile1083 (ABCC1 Tyr1236 [26]), ABCB1 Leu975 [27]), Arg1050 (ABCC2 Arg1210 [30]), Ala1088 (ABCC1 Thr1241 [26]) and Arg1096 (ABCC2 Arg1257 [30]). Altogether, these data confirm that our docking experiments were reliable enough to identify ABCC11 drug binding pockets and suggest that ABCB1, ABCC4, 5 and 11 would share a comparative location for their substrate binding pockets.

### Insight in the impact of Glycine 180 polymorphism

Gly180, found to be inside pocket 2, is prone to G602A SNP (single nucleotide polymorphism) inducing the Gly180Arg mutation which was correlated to earwax type [31], and to colostrum and sweat secretion defaults [32,33]. In addition, ABCC11-Arg180 mutant loses transport capacity toward cGMP [31], in agreement with Gly180 location in our model, in close proximity to the ligand. In the inward-facing model, the Gly180 side-chain points toward phospholipids, while it points toward cGMP in the outward-facing model. Thus, it seems that Gly180 could be in close contact with ligand only in the nucleotide-bound state where the drug-substrate is ready to be released. The altered transport of Gly180Arg-mutant ABCC11 can be explained by the additional charge of arginine versus glycine. This may modify side-chain localization and consequently the binding site conformation. In the outward-facing state, this positive charge could also promote interactions with cGMP and decrease the efficiency of cGMP release into the extracellular space. Toyoda et al. described a default of N-glycosylation proposed to induce protein degradation. [34] Arg180 is thus likely modifying the proper folding of ABCC11 rather than being directly involved in substrate binding.

### Conformational changes revealed by molecular dynamics simulations

**Figure 6:**
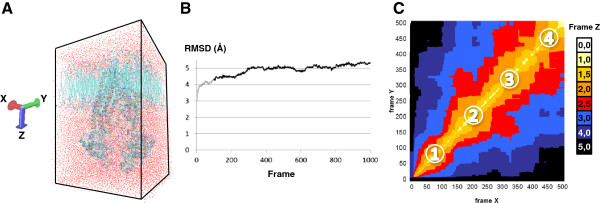
**Structure of the ABCC11 complex for molecular dynamics simulation and RMSD calculations.** (**A**) The inward facing model of ABCC11 was inserted in a phospholipid bilayer membrane surrounded by solvent. (**B**) 1D-RMSD of the ABCC11 complex during the dynamics simulation before (grey) and after (black) reaching equilibrium state. (**C**) 2D-RMSD calculations (X and Y axes represent the frames), carried out on 500 conformations selected with a stride of 2 from the 1000 produced during the indicated time simulation. The four steady-state conformations of each simulation are numbered. Warm colors (yellow and white) revealed steady state was reached.

**Figure 7:**
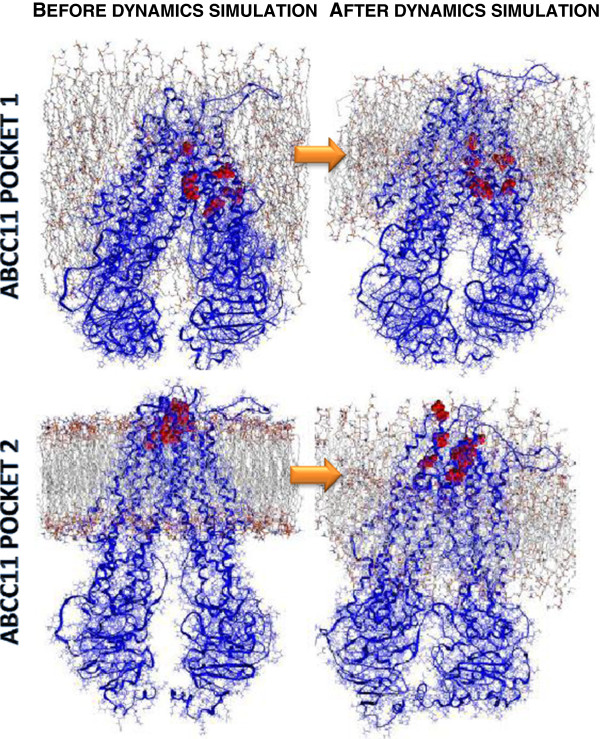
**Molecular dynamics simulation revealed the closing of ABCC11 pocket 1, concomitant with pocket 2 opening.** In order to see their conformation changes, some residues of each pocket are shown in red ball shape within the entire ABCC11 protein. ABCC11 inward-facing conformation is represented by navy blue ribbons with visible amino acid side chains.

We hypothesize that the substrate first binds to the intracellular side of transmembrane region and is then translocated to the extracellular side (subsequent to conformational changes due to ATP binding and hydrolysis). Molecular dynamics simulation was thus performed with the ABCC11 inward facing model inserted into a phospholipid bilayer membrane (Figure 6A). The calculated 1D root mean square deviation (RMSD, based on align seed residue and list distance parameters) on all heavy atoms is weak and evaluated at 3.6944 Å between the frame 100 to frame 1000 (Figure 6B). The 2D-RMSD allowed identifying four principals steady states during these 10 ns dynamics simulation (Figure 6C). Length of the molecular dynamics simulation is limited by the presence of phospholipid membrane and size of the protein (190 KDa). According to the molecular dynamics simulation, the inward-facing model gently changed its conformation. Residues of the internal pocket 1 tended to move closer to each other, suggesting a closing of the bottom part of ABCC11, while residues of the external pocket 2 tended to move away from each other, suggesting by contrast an opening of the top part of ABCC11 (Figure 7). These findings strongly suggested that substrate could enter the transporter from cytoplasm to bind into opened pocket 1 while pocket 2 is closed.

In the literature, Chen et al. described the existence of two different binding sites in ABCC11 for DHEAS and E2-17βG [2]. They proposed that low concentrations of DHEAS had a stimulatory effect on ABCC11 transport of E2-17βG because it would bind to a distinct site, whereas, at high concentration, DHEAS would rather compete with E2-17βG binding at the transport site. Moreover, they observed that E2-17βG exerted an inhibitory effect on DHEAS transport by ABCC11. By consequence, the interaction between E2-17βG and DHEAS is not reciprocal, and do not respond to co-transport situation but rather to the existence of two binding sites. Nevertheless, in the present study, we studied the transport of 5FdUMP and GMPc molecules that are structurally completely different. We suggested that these nucleotide derivatives make different interactions with the residues of ABCC11 binding sites or bind distinct binding sites.

The molecular dynamics simulation of ABCC11 partially simulate the transport cycle of ABCC11. Our data suggested that substrate would cross the protein to bind to pocket 2 (Figure 7). Subsequent conformational changes would open pocket 2 to allow substrate to reach the extracellular space and strongly support the presence of multiple allosteric substrate binding sites in ABCC11 protein. The presence of possible allosteric interactions was supported by Van Aubel’s study [12]. They suggested that ABCC4 transports urate through a positive, cooperative allosteric interaction. Urate changed cGMP transport from an allosteric to a single binding site. Furthermore, they showed that cAMP and cGMP could be simultaneously transported with urate by ABCC4, under saturated conditions, suggesting multiple binding sites. At present, these experimental data are consistent with our hypothesis, which indicate that ABCC11 substrates would bind to a first site and that conformation changes would allow binding to a second site before release and fully explained the 5FdUMP transport process.

## Conclusions

Thanks to homology modeling, we have provided two ABCC11 models corresponding to different catalytic states. We could identify two putative binding pockets for substrates in both models, and recognized several residues that could interact with a bound substrate. These results constitute a useful tool for future biochemical experiments where the specific role of the identified residues could be study *in vitro* by site-directed mutagenesis experiments and transport activity characterization in cell expressing ABCC11. Molecular dynamics simulation strongly helped us to visualize the putative cGMP and 5FdUMP mechanism of secretion. In addition to constitute a complementary tool in identifying the residues’ importance during transport process, they revealed new insights in ABC transporter mechanism of efflux.

## Methods

### Prediction of ABCC11 transmembrane domain (TM)

Data were compiled from TM predictions of 6 independent software analyses: HMMTOP, SOSUI, TMHMM server, TMPRED, TOPRED and PredictProtein. A score was obtained according to the number of predictions for each residue to be located in plasma membrane (Additional file 1). Considering the scale from 1 to 6, residues with a score of at least 4 were considered as belonging to the TM. Nevertheless, although the score of TM12 was only predicted by 3 algorithms, comparison with other ABCC topologies led us to validate this TM12 position.

### Sequence alignments

**Figure 8:**
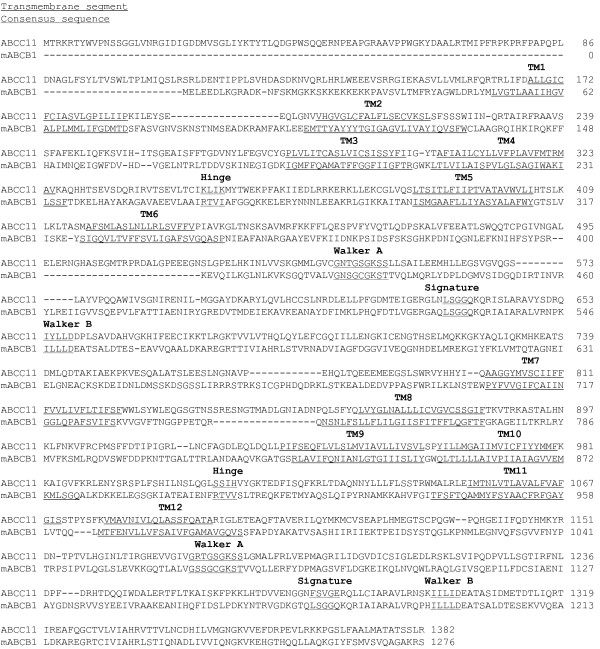
ABCC11 and mABCB1 sequence alignment.

**Figure 9:**
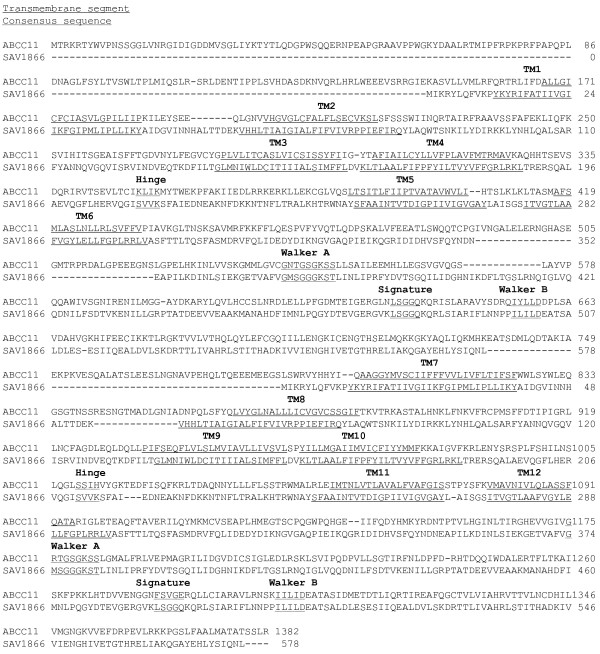
ABCC11 and Sav1866 sequence alignment.

Primary sequences of human ABCC11 (Q96J66), mouse ABCB1 (P21447) and *S. aureus* Sav1866 (Q99T13) were downloaded from the Swiss-prot database. Multiple sequence alignment of amino acid sequences was carried out using ClustalW 2.0. Taking in account TM predictions automatically carried out by softwares, sequence alignment was manually refined, according residue properties (polarity, charge, hydrophobicity) and finally validated to align ABCC11 TM with reference templates (ABCB1 in Figure 8 and Sav1866 in Figure 9). After alignment refinement, the percentage of sequence identity between ABCC11 and mABCB1 was 15.91% while the similarity rose to 29.18% (data obtained from the sequence identity and similarity server, SIAS). With Sav1866, the percentage of sequence identity was 15.93% and the percentage of similarity was 29.1%.

### Selection of X-ray structures and model building

PDB files generated from crystal structures were downloaded from the SWISS-MODEL template library: mouse ABCB1 (PDB code 3G5U) and Sav1866 from *Staphylococcus aureus* (PDB code 2HYD).

Since Sav1866 is a half-transporter (only one MSD and one NBD) and since mouse ABCB1 template was published in two-separate peptides (MSD1-NBD1 and MSD2-NBD2 domains without the flexible linker from residue 627 to 683), the ABCC11 model was generated as two halves: MSD1-NBD1 and MSD2-NBD2 halves. Sequence alignments were submitted to the SWISS-MODEL workspace to generate tri-dimensional models of ABCC11. Minimizations (> 10000 steps with the conjugated gradient algorithm) were carried out with the Sybyl-X 1.1 software package, elaborated by the Tripos company. We applied the Tripos force field with the Gasteiger-Marsilli partial charges and a dielectric constant of 80 to simulate an implicit water phase (the dielectric constant of water is 20.10 at 20°C). No restrains was applied to our models. This step principally refines and corrects the positions of residue side chains.

### Ligand docking

Docking was performed with the Sybyl-X 1.1 software to localize putative binding sites for substrates [35]. Docking simulations were computed using de Surflex-Dock module of the Sybyl X1.1 molecular modeling suite. Charges of molecules were computed using Gasteiger-marsilli algorithm and the option of the docking module were set. The maximum of conformation per fragment was set to 100, to increase possibility of different conformation and the maximal number of rotable bond set to 200. This great number of conformations is allowed because the databank of molecules is small. The following options were selected: soft grid treatment, pre-docking and post-docking minimizations and the molecule fragmentation. Chemscore, D_score, G_score and PMF_score were selected for scoring function. The structure was not relaxed during the scoring calculation.

**Figure 10:**
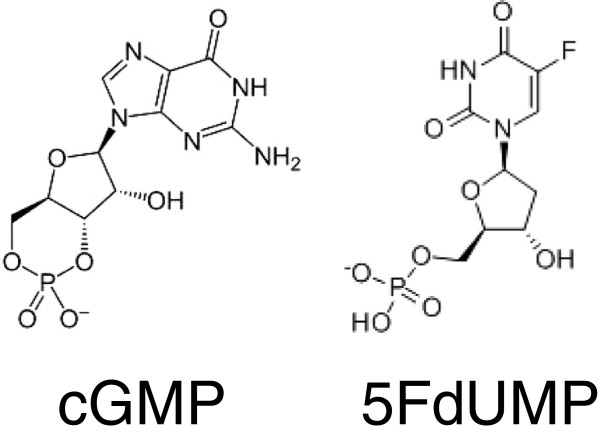
Structures of cGMP and 5FdUMP.

Independent docking runs were carried out for each ligand (cGMP and 5FdUMP, structures in Figure 10). A volume including almost all the entire MSD fraction of ABCC11 was defined in order to identify binding sites either in the transmembrane part or at the membrane leaflet (extracellular or intracellular side). Two main binding pockets were described in each model, for each ligand. In order to refine substrate position, docking runs were performed again by only selecting the amino acid residues constituting those pockets.

### Molecular dynamics simulation

The model of the ABCC11 protein was loaded in VMD 1.8.3 program. A POPC phospholipid bilayer was added with the membrane builder module. The ABCC11 protein was inserted in the membrane according to known area [36,37]. All POPC in contact with the protein were removed and the resulting model was inserted in a parallelepipedic TIP3P solvent box with the add solvation box module of VMD 1.8.3 software. A distance of 15 Å was set between the surfaces of the protein to the limit of the solvent box; the resulting models have a membrane size of 94 Å × 97 Å with a height of 135 Å.

Conditions were computed to reach neutral charges before adding sodium and chloride to concentrations corresponding to physiological conditions. The model was minimized with the NaMD 2.8 b1 software for 1000 steps before the molecular dynamics simulations [38]. It was computed on a 144 xeon core CPU cluster supercomputer (SGI Altix).

Simulations were carried out at constant temperature (300 K) and pressure (1 atm) and by implementing the widely used CHARMM 27 force fields. The time step was set at 1 fs and Langevin dynamics was performed with a target piston pressure of 1.01325 bar and a damping coefficient of 5 ps^-1^. There is no coupling of the Langevin temperature with hydrogen. The PME algorithms were applied with a grid extended by 10 Å from the PBC size [39]. The electrostatic cut-off was set at 14 Å. A conformation was sampled every 10 ps. As the solvent was described, the dielectric constant was set at 1.

To identify steady conformations, 2D-RMSD calculations were carried out on 500 conformations selected, with a stride of 2, from the 1000 conformations produced during the 10-ns simulation. The equilibrium state was reached around 1 ns for studied model, and is longer than usual as it includes phospholipid membrane.

## Abbreviations

5FdUMP: 5′-fluoro-2′-deoxyuridine monophosphate; ABC: ATP-binding cassette; ECL: Extracellular loop; ICL: Intracellular loop; MDR: MultiDrug resistance; MRP: Multidrug resistance protein; MSD: Membrane spanning domain; NBD: Nucleotide-binding domain; RMSD: Root mean square deviation; TM: Transmembrane segment.

## Competing interests

The authors declare that they have no competing interests.

## Authors’ contributions

MH carried out transmembrane segment prediction, sequence alignments, model building and docking analyses, and wrote the manuscript. RT participated in model minimization, carried out molecular dynamics simulation, and drafted the manuscript resulting parts. PF participated in sequence alignments and model building, and revised the manuscript. ADP participated in revising the manuscript critically for important intellectual content. CD participated in revising the manuscript. LP conceived the study, and participated in its design and coordination and wrote the manuscript. All authors read and approved the final manuscript.

## Supplementary Material

Additional file 1 **Predictions of amino acid residues located inside the membrane.** TransMembrane segments (TMs) are numbered from 1 to 12. The positions of the first and last amino acid residues are indicated in the first column. The second line reports the prediction score obtained by independent softwares: HMMTOP, SOSUI, TMHMM server, TMPRED, TOPRED and PredictProtein. Click here for file
