# Supplementary figures and images for: Localization of putative binding sites for cyclic guanosine monophosphate and the anti-cancer drug 5-fluoro-2′-deoxyuridine-5′-monophosphate on ABCC11 in silico models
Source: BMC Struct Biol. 2013 May 6;13:7. doi: 10.1186/1472-6807-13-7 (PMC3668285; doi:10.1186/1472-6807-13-7)

## Slide 1
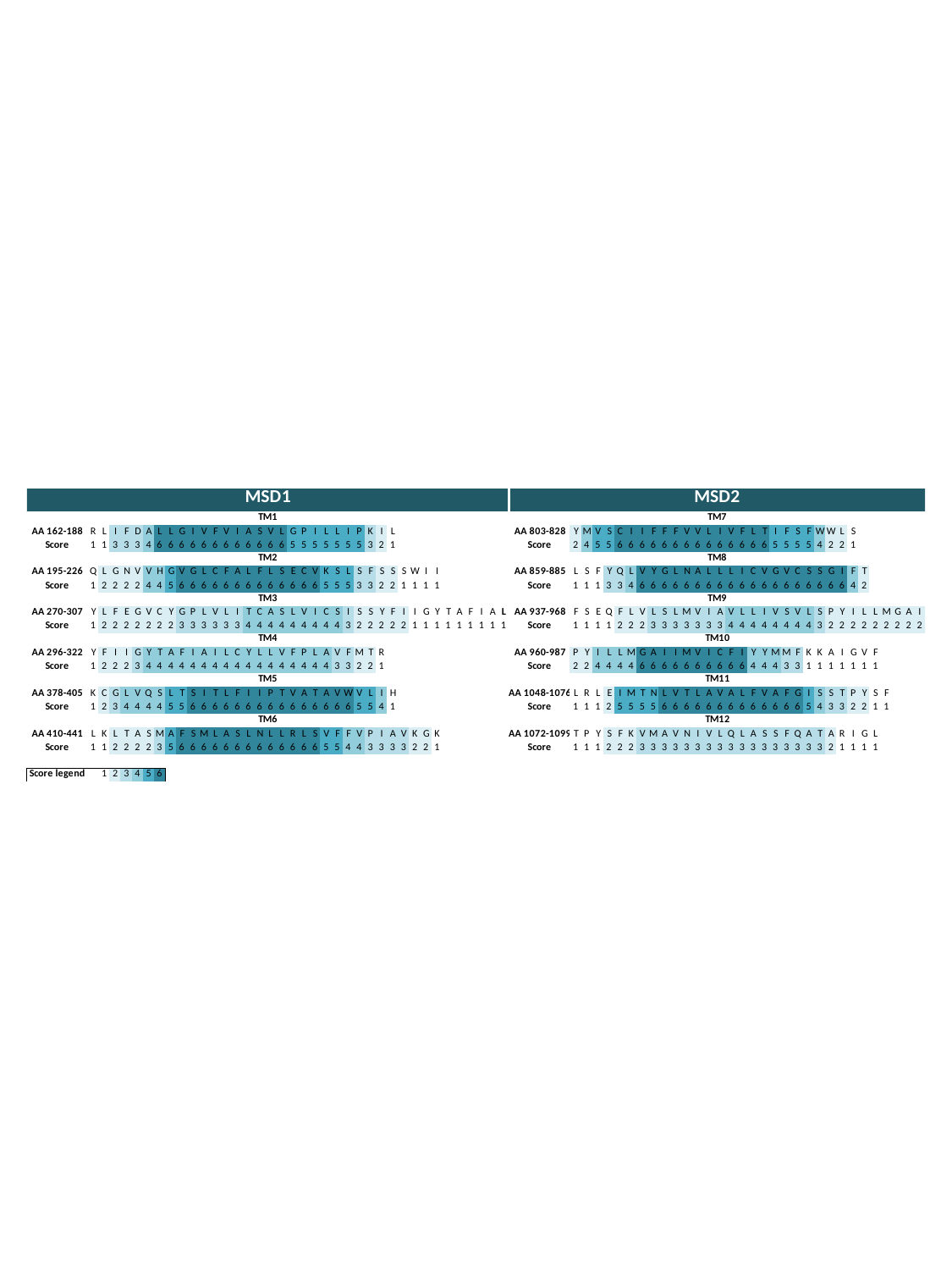

Supplement: Additional file 1 — Predictions of amino acid residues located inside the membrane. TransMembrane segments (TMs) are numbered from 1 to 12. The positions of the first and last amino acid residues are indicated in the first column. The second line reports the prediction score obtained by independent softwares: HMMTOP, SOSUI, TMHMM server, TMPRED, TOPRED and PredictProtein. [file 1472-6807-13-7-S1.pptx]
